# Effect of Signal Peptide on Stability and Folding of *Escherichia coli* Thioredoxin

**DOI:** 10.1371/journal.pone.0063442

**Published:** 2013-05-07

**Authors:** Pranveer Singh, Likhesh Sharma, S. Rajendra Kulothungan, Bharat V. Adkar, Ravindra Singh Prajapati, P. Shaik Syed Ali, Beena Krishnan, Raghavan Varadarajan

**Affiliations:** 1 Molecular Biophysics Unit, Indian Institute of Science, Bangalore, India; 2 Chemical Biology Unit, Jawaharlal NehruCentre for Advanced Scientific Research, Jakkur, Bangalore, India; Aligarh Muslim University, India

## Abstract

The signal peptide plays a key role in targeting and membrane insertion of secretory and membrane proteins in both prokaryotes and eukaryotes. In *E. coli,* recombinant proteins can be targeted to the periplasmic space by fusing naturally occurring signal sequences to their N-terminus. The model protein thioredoxin was fused at its N-terminus with malE and pelB signal sequences. While WT and the pelB fusion are soluble when expressed, the malE fusion was targeted to inclusion bodies and was refolded *in vitro* to yield a monomeric product with identical secondary structure to WT thioredoxin. The purified recombinant proteins were studied with respect to their thermodynamic stability, aggregation propensity and activity, and compared with wild type thioredoxin, without a signal sequence. The presence of signal sequences leads to thermodynamic destabilization, reduces the activity and increases the aggregation propensity, with malE having much larger effects than pelB. These studies show that besides acting as address labels, signal sequences can modulate protein stability and aggregation in a sequence dependent manner.

## Introduction

In *E. coli* two distinct pathways exist for the export of proteins across the cytoplasmic membrane. The majority of protein translocation across the cytoplasmic membrane occurs in the unfolded state via the Sec translocon [1], [2]. Another pathway is the *t*win-*a*rginine *t*ranslocation (Tat) pathway, so called because of the signature Arg-Arg motif found near the N terminus of the leader peptide of proteins that are engaged in this mode of export from the cytoplasm. Tat pathway translocates folded proteins post-translationally via the Tat translocon [3], [4].

Proteins which are exported through the Sec translocon contain a hydrophobic signal sequence at their N termini [5]. Translocation can be co-translational or post-translational. The former pathway is used for proteins with hydrophobic transmembrane segments or secreted proteins which have more hydrophobic signal sequences than those employed in the latter, post-translational pathway [6]. In the co-translational pathway, upon emerging from the ribosome, the N-terminal signal sequence binds to the signal recognition particle (SRP). Following interaction with the membrane receptor FtsY, the complex of nascent chain and ribosome is transferred to the SecYEG translocase. In the latter post-translational pathway, upon emerging from the ribosome, proteins bind first to trigger factor, then SecB and SecA. Binding of SecA bound preproteins to the SecYEG translocase initiates the process of translocation [7], [8]. Prior to translocation, pre-proteins must be maintained in an export competent conformation in the cytoplasm which is thought to be a loosely folded, protease-sensitive structure [9]. The export competent conformation is maintained by chaperone proteins SecB, GroEL, DnaK, and DnaJ, which also aid in preventing aggregation and improper intramolecular interactions of the exported proteins [10]–[15]. In addition, the presence of non-optimal codons in the signal sequence have been shown to play an important role in export for both SecB and SRP dependent export [16], [17]. Recombinant proteins in *E. coli* can be targeted to the periplasmic space via the Sec-dependent pathway by fusing naturally occurring signal sequences to their N-terminus. Signal sequences can also be present within a protein or at its C-terminal end. For some proteins, periplasmic expression is advantageous over cytoplasmic production in a number of ways. For instance, an authentic N-terminus devoid of an N-terminal Methionine can be obtained after removal of signal sequences by leader peptidases. The periplasm is also conducive to disulfide formation, has fewer proteases compared to the cytoplasm and many have their own specific substrates. Finally, there are fewer proteins in the periplasm than the cytoplasm and its content can be selectively released by osmotic shock or other strategies thereby facilitating protein purification [18], [19].

The signal peptide plays a key role in targeting and membrane insertion of secretory and membrane proteins in both prokaryotes and eukaryotes [20], [21]. After membrane insertion, signal sequences are cleaved off by the membrane bound signal peptidases. Signal sequences have a common tripartite structure consisting of a positively charged N-terminus (N-region), a stretch of 7–15 hydrophobic residues (H-region) and a more polar region containing helix breaking Proline and Glycine residues as well as the signal peptide cleavage site (C-region) [5], [22].

*E. coli* signal sequences are distinct for proteins that are periplasmic, inner and outer membrane-bound, and secreted outside the cell [23]. Sequence differences are also observed between mycoplasmas and other gram-negatives such as *E. coli* [24]. *E. coli* signal peptides can replace the native signal peptide of heterologous proteins for efficient expression in *E. coli* [25].

We have previously shown that the maltose-binding protein containing its native the N-terminal 26-residue malE signal peptide is substantially less stable and more aggregation prone than the corresponding mature protein [14], [15]. We now explore the effects of two different signal peptides, pelB and malE on protein stability and aggregation in a smaller protein, *E. coli* thioredoxin. pelB refers to the 22 N-terminal leader sequence of pectatelyase B of *Erwinia carotovora* CE [26].The pelB leader sequence when attached to a protein, directs the protein to the bacterial periplasm, where the sequence is removed by a signal peptidase. pelB has been used to direct the coat protein-antigen fusions to the cell surface in engineered bacteriophages used for the purpose of phage display [27]. Both pelB and malE signal peptides utilize the post-translational translocation pathway. This has been experimentally shown for malE [28], [29] and inferred for pelB based on the hydrophobicity of the signal sequence [27].

*E. coli* thioredoxin is a 108 amino acid long, heat stable and redox active polypeptide containing 2 cysteine, 5 proline and 2 tryptophan residues [30] whose folding pathway has been well characterized. The two tryptophans are at positions 28 and 31.Trp 31 is conserved throughout the known thioredoxin sequences, while Trp28, is conserved only in prokaryotes and is replaced by serine in eukaryotes [31]. The active site disulfide is located close to the two tryptophans in the sequence (Trp28-Ala-Glu-Trp31-Cys32-Gly-Pro-Cys35-) as well as in the three dimensional structure [32]–[34]. This proximity results in strong quenching of tryptophan fluorescence in the native protein that is relieved upon reduction of the disulfide or denaturation of the protein [35], [36]. The single disulfide bond bridges the first and fourth residue of a type III reverse turn involving residues [32]–[35] that likely persists in the denatured protein and could in principle direct the folding of polypeptide into its native confirmation [36], [37]. Thioredoxin has also been used as a fusion partner to facilitate folding of other proteins [37], [38]. Thioredoxin is well characterized in terms of its structure, stability and folding. For these reasons it is a useful model system to study the effects of signal peptides on protein stability and folding. In the present study, we compare properties of WT Trx with those of fusions of Trx with signal peptides of malE (malE Trx) and pelB (pelB Trx) respectively.

## Materials and Methods

### Plasmids Used

Two leader sequences involved in directing proteins to the periplasmic space, pelB and malE were used for the experimental studies. These sequences are indicated below.

*MKYLLP* TA**E**AGLLLLLA APQIA (pelB).

*MKKTGAR* ILALS**E**LTTM MFSASALA (malE).

Here, the positively charged N-region, hydrophobic central region and the polar C-region are shown in italics, underlined and normal text respectively. They were assigned as described [39]. The A to E mutation in each sequence introduced to prevent translocation and signal peptide cleavage is highlighted in bold.

pelB (A9E)Trx and malE (A14E)Trx fusions were constructed by overlap PCR and cloned into pET22b(+) and pET20b(+) vectors respectively between the *Nde*I and *Hind*III sites.

### Protein Expression and Purification

WT Trx and its derivatives were transformed into *E. coli* BL21 (DE3) cells and expressed under the control of the T7 promoter at 37°C. WT Trx was isolated following chloroform shock and purified using Q-Sepharose ion-exchange chromatography as described previously [40]. The cells were grown in 1L of Luria-Broth (LB) at 37°C to OD_600_ = 0.6, induced with 0.4 mM IPTG and pelleted by centrifugation after 4 hours. Chloroform was added to the resuspended pellet in an equal volume and incubated at room temperature under shaking condition. This was followed by addition of 100 ml of buffer (20 mM Tris, 25 mM NaCl, pH 7.4). The resulting mixture was spun at 4000 rpm at 4°C to separate the chloroform layer from the aqueous layer. The aqueous layer was loaded onto a Q-Sepharose column pre-equilibrated with 20 mM Tris (pH 7.4) followed by washing with 20 mM Tris, 25 mM NaCl (pH 7.4). A linear gradient of 25–500 mM NaCl in 20 mM Tris, pH 7.4 was used to elute the protein. Thioredoxin elutes between 100 and 125 mM NaCl. Purified protein fractions were dialyzed into CGH-10 buffer (10 mM each of citrate, glycine, and HEPES) (pH 7.4) and concentrated to a final concentration of 6 mg/ml. pelB Trx was purified from the soluble lysate in a similar fashion on a Q-Sepharose column, following cell lysis by sonication. The proteins were dialyzed and concentrated to a final concentration of 15 mg/ml. As malE Trx was insoluble, it was refolded from inclusion bodies. A cell pellet containing malE Trx was sonicated in Tris buffer (25 mM, pH 8.0) containing 0.1 mM PMSF +0.5% Triton X-100 and spun at 14,000 rpm for 30 min. The resulting pellet was resuspended in the same buffer (without PMSF) by sonication (30 s pulse on time) and centrifuged again at 14,000 rpm for 30 min. To this pellet, 6.0 M GdmCl in Tris buffer (50 mM, pH 8.0) containing 2 mM DTT was added and the solution was stirred overnight. After removing insoluble material by centrifugation, the protein was refolded by 10-fold rapid dilution into Tris buffer (50 mM, pH 7.4). Refolding was carried out at 4°C. Insoluble aggregates were removed by centrifugation and the protein was dialyzed and concentrated to a final concentration of 4 mg/ml. Tricine-PAGE confirmed the purity of proteins. Purified proteins were stored in aliquots at −70°C.

### Mass Spectrometry

Prior to mass spectrometry, protein samples were desalted into water using a PD-10 column (GE Healthcare). ESI-MS was performed on a Micro mass machine in positive ion mode with the desolvation temperature set at 150°C.

### Analytical Gel Filtration

Proteins were subjected to gel filtration chromatography using an analytical Superdex–75 (GE Healthcare, Column volume, V_t_ = 24 ml, void volume = 8 ml) column on a Duo Flow FPLC system (BioRad). The column was equilibrated with CGH-10 buffer, pH7.4. 25–40 µg of protein in 250 µl was loaded on to the column and eluted at a flow rate of 0.4 ml/min.

### CD Measurements

Far UV CD spectra were acquired on a JASCO J715 spectropolarimeter. A protein concentration of 10 µM was used in a 1 mm path length quartz cuvette. Measurements were done at 25°C over a wavelength range of 200 to 250 nm at a scan rate of 50 nm/min. Data was collected with response time of 4 s, 2 nm bandwidth and an average of four scans. Buffer scans were acquired under similar conditions and subtracted from the protein spectrum before analysis. Mean residue ellipticity was calculated as described [41].

Near UV CD spectra were acquired on a JASCO J715 spectropolarimeter. Protein concentrations of 600 µM, 400 µM and 250 µM for WT Trx, pelB Trx, and malE Trx respectively were used in a 2 mm path length quartz cuvette. Measurements were done at 25°C, over a wavelength range of 250 to 300 nm, at a scan rate of 10 nm/min. Data was collected with response time of 8 s, bandwidth of 2 nm and an average of four scans was taken. Buffer scans were acquired under similar conditions and subtracted from the protein spectrum before analysis. Mean residue ellipticity was calculated as described [41].

### Fluorescence Measurements

ANS fluorescence measurements were done at 25°C on a JASCO FP-6300 spectrofluorometer in a 1 cm water-jacketed cell using an excitation wavelength of 388 nm and an emission wavelength range of 420–550 nm. Spectra were averaged over four consecutive scans. As a positive control, a previously characterised molten globule of maltose-binding protein (MBP) at pH 2.5 was used [42].

### Proteolytic Digestion (WT Trx, pelB Trx and malE Trx)

Controlled proteolysis was carried out in CGH-10 buffer, pH 7.4 containing 150 mM sodium chloride at 37°C using the proteases Papain and Proteinase K. The enzyme:substrate ratio used was 2% (w/w). Final protein concentrations used in the reaction were 1 mg/ml, 1 mg/ml and 0.5 mg/ml for WT Trx, pelB Trx and malE Trx respectively. The proteolytic mixture was kept for either 0 minute (control) or for 30minutes at 37°C. The reaction was quenched with 1 µM Iodoacetic acid (Papain) and 5 µM Phenylmethanesulfonic acid, PMSF (Proteinase K). Subsequently, SDS-PAGE loading dye was added (2% SDS, 0.1% Bromophenol Blue, 10% Glycerol and 5% β-mercaptoethanol). Samples were subjected to 15% Tricine-PAGE after boiling for 10 min followed by staining in Coomassie Brilliant Blue R250.

### Isothermal GdmCl Denaturation Studies

Chemical denaturation studies were carried out by monitoring the Trp fluorescence signal at 340 nm in a JASCO FP-6300 spectropolarimeter, using an excitation wavelength of 280 nm. Excitation and emission bandwidth used were 2.5 nm and 5 nm respectively. Protein concentrations used were 12 µM, 16 µM and 16 µM for pelB Trx, WT Trx and malE Trx respectively. Proteins were incubated in different concentrations (0 to 6 M) of GdmCl in CGH-10 buffer (pH 7.4) at 25°C, overnight for equilibrium to be established prior to fluorescence measurement. Data were fit to a two-state N to U model [43].

### Thermal Denaturation Studies

Thermodynamic parameters for thermal denaturation were measured by differential scanning calorimetry (DSC). Proteins (0.2 mg/ml) were subjected to a thermal gradient from 30°C to 110°C at a scan rate of 60°C/h in CGH-10 buffer (pH 7.4). DSC for malE Trx at a higher concentration (2.5 mg/ml) was also carried out. Data were analyzed as described previously [41].

### Refolding in Buffer

The proteins were denatured in 6 M GdmCl for at least 6 to 8 hours and refolding was initiated by rapid dilution (10-fold) into CGH-10 buffer (pH 7.4). Refolding was measured by monitoring the intrinsic Trp fluorescence. The spectra were obtained after 1 hour over a range of 300 nm to 400 nm using an excitation wavelength of 280 nm. The emission and excitation bandwidths used were 5 and 2.5 nm respectively. Protein concentrations used for CD and fluorescence were 10 µM and 5 µM respectively.

### Insulin Reduction Assay

The activity of the Trx proteins was assessed by the insulin reduction assay described by Holmgren [44]. Protein concentration used was 5 μΜ.

### Refolding in the Presence of Crowding Agent

The proteins were denatured in 4 M GdmCl in CGH-10 (pH 7.4) for about 6 hours before refolding studies were carried out. Aggregation propensity of the proteins was studied by following the scattering at 320 nm during refolding. Refolding was initiated by 10-fold rapid dilution of 100 µM denatured proteins in 4 M GdmCl (CGH-10 buffer, pH 7.4) into CGH-10 buffer (pH 7.4) without or with the crowding agent, 30% Ficoll-70. Proteins were refolded to final concentrations of 2, 5, 7.5 and 10 µM.

### Analysis of Signal Peptide Aggregation Propensities

Aggregation propensity profiles for various signal sequences were calculated using three different servers, namely Zyggregator [45], PASTA [46], AGGRESCAN [47]. The Zyggregator server outputs the aggregation propensity score, Z_agg_ for every amino acid in the query protein sequence. All the calculations were done at pH 7.4. A stretch of amino acid sequence having Z_agg_>1 is considered to have high aggregation propensity. The region with Zagg<0 is considered to have low propensity to aggregate. The PASTA server gives the per-residue aggregation probability, *h*(k).The regions with high *h*(k) values are considered to be involved in intermolecular pairing, resulting in aggregation. The amyloidogenic regions of the human amyloid β-peptide (Aβ-40) possess *h*(k) values in the range of 0.05–0.06. AGGRESCAN predicts hot-spots of aggregation in the query amino acid sequence based on a propensity scale derived from in vivo aggregation experiments. The amino acids with propensity values greater than −0.02 are considered as hot-spots of aggregation. Window width of 5 was used for these calculations. In addition, average hydrophobicity of the query amino acids, as a possible probe for aggregation, was also calculated using the program PREDBUR [48]. The width of the sliding window was set to 7. All the four algorithms were applied to the pelB and malE signal sequences. To assess the prediction accuracies of Z_agg_, a control set of phoA, treA, and pcoE signal peptides, previously studied as soluble Trx fusion systems, was also used.

## Results

### Protein Expression

The malE A14E mutation has been described previously [49] and contains an A to E mutation in the hydrophobic central region of the signal peptide. This mutation renders the signal peptide export defective. We therefore hypothesized that a similar mutation in the pelB signal sequence (A9E) would also have a similar effect. To confirm this hypothesis the variants of Trx having mutations in the leader sequences, pelB Trx and malE Trx were cloned into vector pET22b(+) and pET20b(+) respectively. pelB Trx and malE Trx contains mutations A9E and A14E in pelB and malE signal sequences respectively, to prevent translocation and signal peptide cleavage of the corresponding Trx fusions. Although, Trx is a cytoplasmic protein, it is released into the periplasm following chloroform shock [50]. WT Trx and pelB Trx could be isolated in soluble form and purified on a Q Sepharose column with yields of 70 mg/l and 20 mg/l respectively. malE Trx was refolded from inclusion bodies with a yield of 10–15 mg/l. As expected signal sequence remain uncleaved in pelB Trx and malE Trx. Tricine-PAGE confirmed the purity of the proteins. Molecular weights of the proteins were determined accurately by ESI-MS. Representative ESI-MS traces are shown in Figure S1A-C. For WT Trx, the expected and observed MW’s are 11673.3 and 11672.8 Da respectively (Figure S1A). Both signal peptide containing proteins, pelB Trx and malE Trx contain intact signal peptide. For pelB Trx, the expected and observed MW’s are 14075 Da and 14073.5 Da (Figure S1B), while for malE Trx the expected and observed MW’s are 14571Da and 14573.8 Da respectively (Figure S1C).

### Analytical Gel Filtration

**Figure 1:**
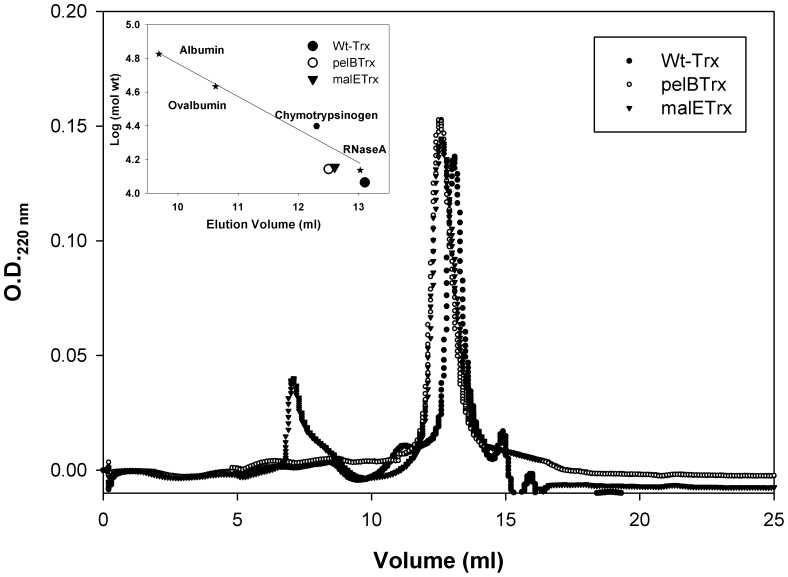
Superdex 75 gel filtration elution profiles for Thioredoxin derivatives. Profile indicates that all proteins are monomeric. The inset shows the calibration curve using the marker proteins albumin, ovalbumin, chymotrypsinogen and ribonucleaseA with molecular weights of 67, 43, 24 and 13 kDa respectively. pelB Trx and malE Trx have identical elution profiles. The gel filtration column used was an analytical Superdex–75 (GE Healthcare, Column volume, V_t_ = 24 ml, void volume = 8 ml) column.

The aggregation state of the proteins was examined by performing gel filtration studies. The elution volumes were 13.1 ml for WT Trx and 12.5 ml for both pelB Trx and malE Trx. malE Trx and pelB Trx elute between chymotrypsinogen and RNaseA and appear to be monomeric (Figure 1).

### CD Measurements

**Figure 2:**
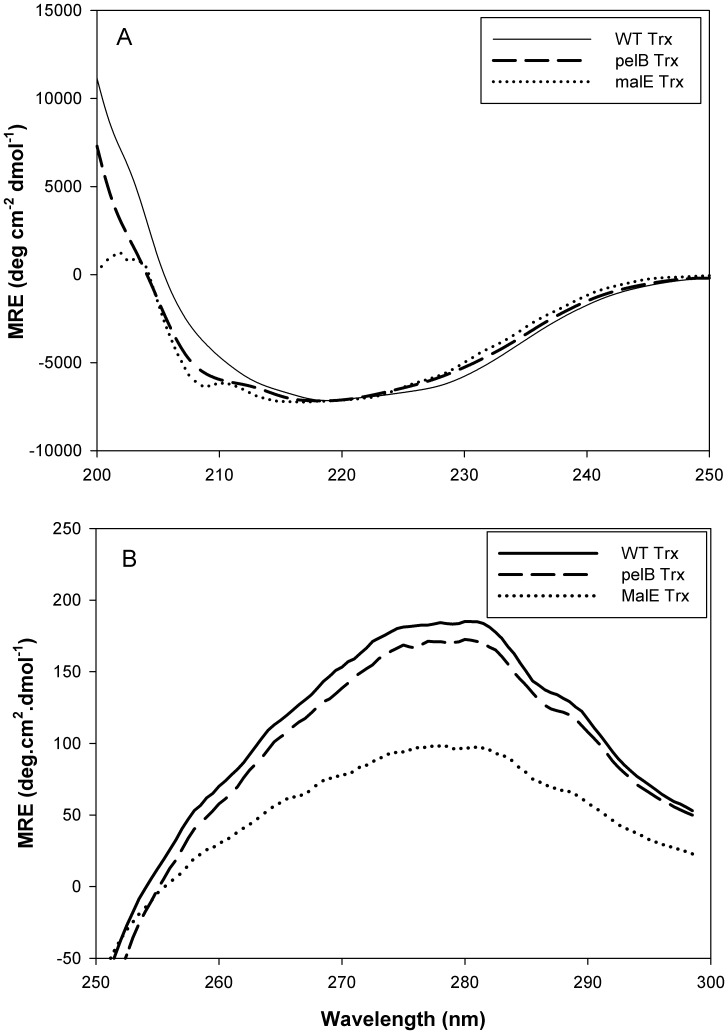
CD spectra of WT Trx (−), pelB Trx (–) and malE Trx (···).(A) Far UV CD spectra of WT Trx (-), pelB Trx (–) and malE Trx (···) were obtained with 10 µM protein solution in CGH-10 buffer, pH 7.4 at 25°C with a 0.1 cm path-length cuvette. (B) Near UV CD spectra were obtained using protein concentrations of 600 µM, 400 µM and 250 µM for WT Trx, pelB Trx, and malE Trx respectively. Measurements were done in CGH-10 buffer, pH 7.4 at 25°C with a 0.2 cm path-length cuvette.

Far UV CD (200–250 nm) serves as a good reporter of the secondary structural contents of proteins under different conditions [51]. The mean residue ellipticity (MRE) in the far UV range as a function of wavelength is shown in Figure 2 quantitatively, MRE values are similar for all the three proteins. From WT Trx to pelB Trx and malE Trx little structural change is observed though pelB Trx and malE Trx show a slight additional dip at 210 nm (Figure 2A).

Near UV CD (200–250 nm) serves as reporter of the tertiary structure of proteins. The mean residue ellipticity (MRE) in the Near UV range as a function of wavelength is shown in Figure 2B. Quantitatively, MRE values are similar for WT Trx and pelB Trx but malE Trx shows somewhat less structure than WT Trx and pelB Trx.

### ANS Binding

**Figure 3:**
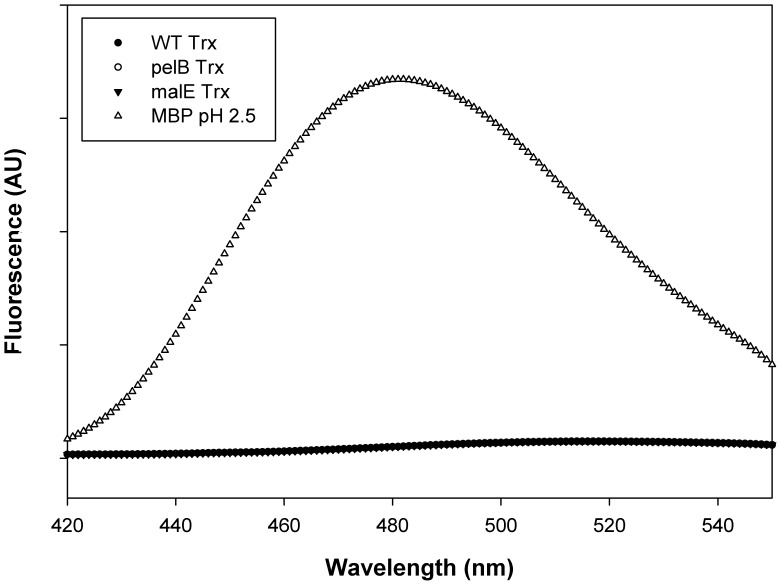
ANS Fluorescence emission spectra. The data points are shown as closed circle (•) for WT Trx, open circle (○) for pelB Trx, closed triangle (▾) for malE Trx, and open triangle (Δ) for MBP. Curves for WT Trx, pelB Trx and malE Trx are not distinguishable due to overlapping data. Protein concentration was 3 µM and ANS concentration was 300 µM. Sample excitation was at 388 nm. All Trx spectra were acquired in CGH 10 buffer pH 7.4 and the MBP spectrum was acquired in CGH 10 buffer pH 2.5.

ANS binding of the proteins was monitored at pH 7.4 for WT Trx, pelB Trx and malE Trx (Figure 3). As compared to the control molten globule of MBP at pH 2.5, none of the proteins show binding to ANS which indicates an absence of molten globule formation at neutral pH.

### Proteolytic Digestion

**Figure 4:**
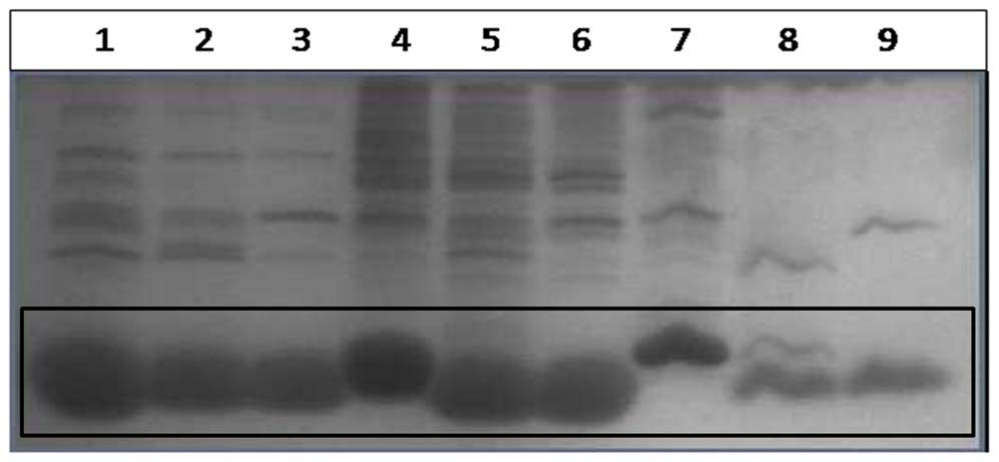
Tricine-PAGE analysis of proteolytic digests of WT Trx, pelB Trx and malE Trx. These were performed at 37°C for 30 min and demonstrate that the signal peptides are protease accessible. Lanes 1–3 show undigested, papain and Proteinase K digested WT Trx. Lanes 4–6 show undigested, papain and Proteinase K digested pelB Trx. Lanes 7–9 show undigested, papain and Proteinase K digested malE Trx respectively. Proteolysis was stopped after 30 min by the addition of 1 µM Iodoacetic acid for Papain and 5 µM Phenylmethanesulfonic acid (PMSF) for Proteinase K. Samples were boiled with SDS-PAGE gel loading dye (2% SDS, 0.1% bromophenol blue, 10% Glycerol and 5% β-mercaptoethanol) prior to loading on the gel. Following electrophoresis, proteins were visualized by staining with Coomassie brilliant Blue R250. The relevant bands are enclosed by a box.

Proteolysis was carried out using Proteinase K and Papain for 30 minutes at 37°C. Under these conditions, the signal peptide in pelB Trx and malE Trx is rapidly cleaved. Following cleavage of signal peptide, pelB Trx and malE Trx which now lack the major portion of signal peptide are resistant to further digestion like WT Trx (Figure 4). This suggests that in the folded state, the signal peptide is accessible to proteases and does not significantly perturb the overall structure of the protein.

### Isothermal GdmCl Denaturation Studies for WT Trx, pelB Trx and malE Trx

**Figure 5:**
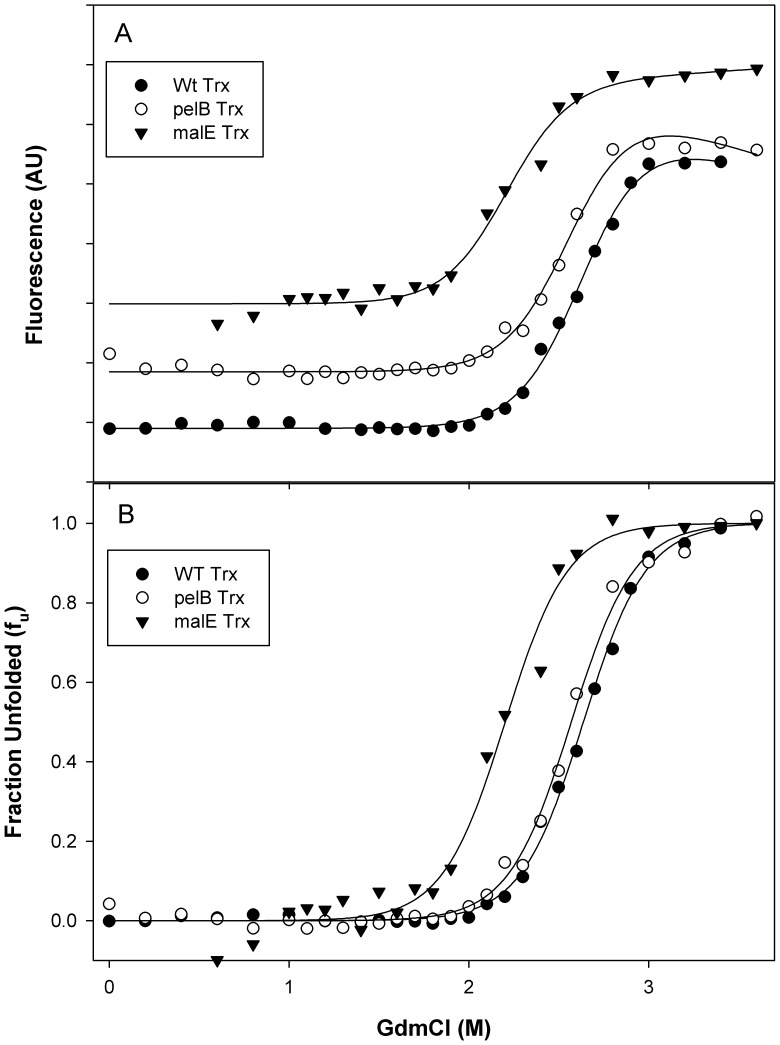
Isothermal GdmCl denaturation. Isothermal GdmCl denaturation studies were carried out at 25°C for WT Trx (○), pelB Trx (•) and malE Trx (▾) in CGH-10 buffer (pH 7.4) (A) Fluorescence change as a function of GdmCl concentration. The continuous line through the data is a fit of the data to a two-state unfolding model. (B) Fraction unfolded (f_u_) as a function of denaturant concentration. The continuous line through the data is a fit of the data to a two-state unfolding model, using a m-value of 3.40 kcal mol^−1^ M^−1^. Values of ΔG° were 8.9±0.1 and 8.7±0.1 kcal mol^−1^7.5±0.1 kcal mol^−1^for WT Trx, pelB Trx and malE Trx respectively. Corresponding C_m_ values were 2.60, 2.5 M and 2.2M respectively.

The unfolding transition was monitored using the Trp fluorescence signal at 340 nm (Figure 5A). The continuous solid line through the data is a fit to a two-state NU model (Figure 5A and 5B) [43] and yields a C_m_ of 2.60 M for WT Trx, 2.50 M for pelB Trx and 2.20 M for malE Trx. The free energies of unfolding at zero denaturant concentration (ΔG°) were found to be 8.9±0.1, 8.7±0.1 and 7.5±0.1 kcal/mol for WT Trx, pelB Trx and malE Trx, respectively. Similar values of unfolding free energies have been previously determined for WT Trx [52], [53]. A similar destabilization of malE MBP relative to mature MBP lacking the malE signal peptide was seen previously [49].

### Thermal Denaturation Studies

**Figure 6:**
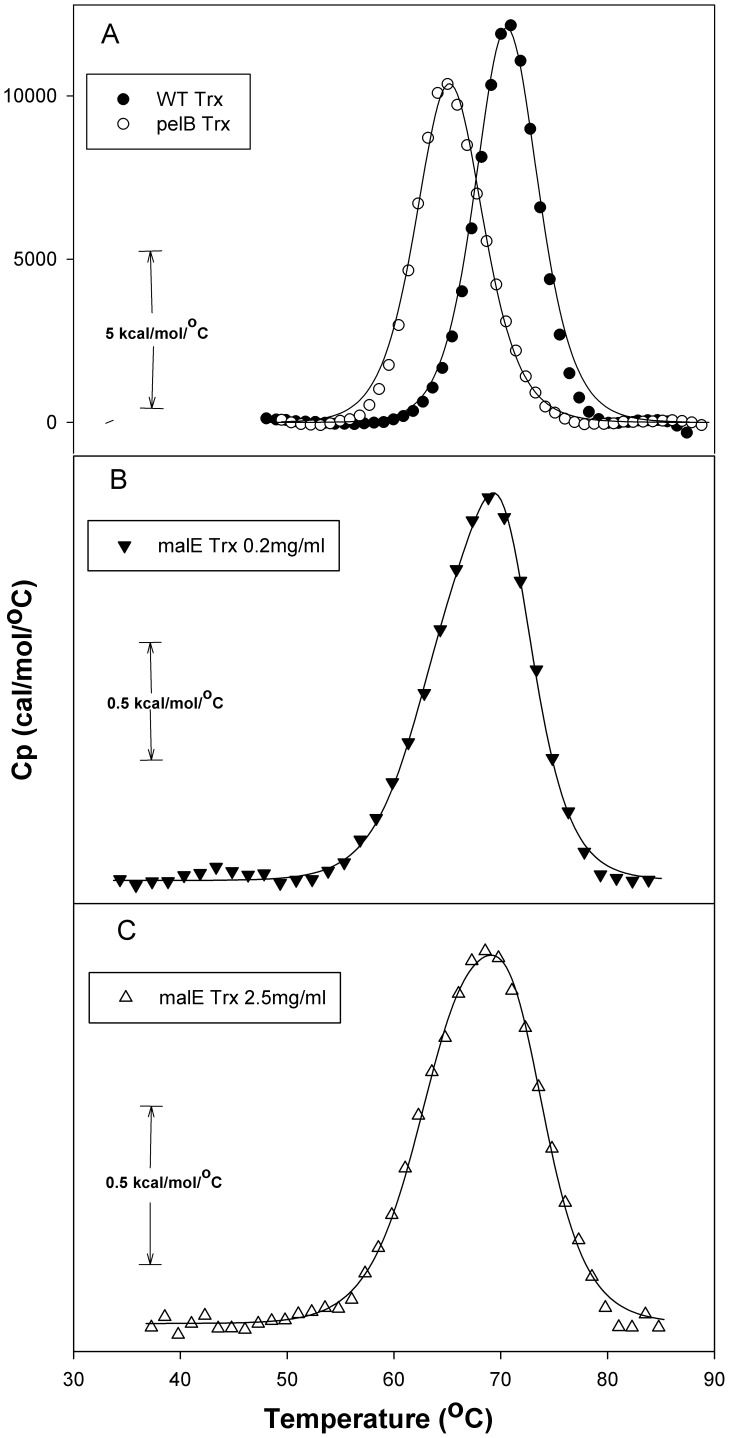
Representative DSC scans of WT Trx, pelB Trx and malE Trx. Scans were carried out in CGH-10 buffer (pH 7.4). The scan rate was 60°C /h and protein concentration was 0.2 mg/ml. Baseline subtracted excess heat capacity data as a function of temperature are shown. The data indicate that protein stability increases in the order malE Trx<pelB Trx<WT Trx. The data points are shown as closed circle (•) for WT Trx, open circle (○) for pelB Trx and closed triangle (▾) for malE Trx, all at 0.2 mg/ml concentration and open triangle (Δ) for malE Trx at a concentration of 2.5 mg/ml (A) Representative DSC scans of WT Trx and pelB Trx, the line shows the fitting to a two state model with one peak. (B) Representative DSC scans of malE Trx at 0.2 mg/ml concentration, the line shows the fitting to non-two state model with a single peak.(C) Representative DSC scans of malE Trx at 2.5 mg/ml concentration; the line shows the fitting to a non-two-state with a single peak.

The energetics of thermal unfolding was characterized by examining the thermal stability of each protein employing high-sensitivity differential scanning calorimetric (DSC) measurements. Figure 6 shows buffer-corrected partial molar excess heat capacity data. The thermal stability (T_m_) of pelB Trx was lower by ∼6°C compared to WT Trx (Figure 6A and Table 1), while malE Trx shows considerably lower thermal stability with an unfolding transition at 72 °C (Figure 6B and Table 1). While thermal unfolding of WT Trx and pelB Trx were reversible and two-state, thermal unfolding of malE Trx was completely irreversible and non-two-state, suggesting that unfolding is followed or accompanied by aggregation. When thermal unfolding of malE Trx was carried out at an approximately tenfold higher concentration (Figure 6C) there is an observable decrease in the thermal stability (of ∼4°C) and broadening of the transition (Figure 6C and Table 1), also consistent with increased aggregation at higher concentrations of malE Trx. Since the thermal unfolding at both the concentrations is irreversible, detailed thermodynamic analysis is difficult. However, the lower thermal stability of malE Trx is clear from the data.

**Table 1:** Thermal denaturation parameters at pH 7.4 for WT Trx, pelB Trx and malE Trx obtained from DSC.

Protein	ΔH° (T_m_) (kcal/mol)(kcal/mol)	T_m_ (^o^C)
WT Trx	112±0.41	87.3±0.03
pelB Trx	102±0.31	81.1±0.02
malE Trx^a^	16±0.15	71.9±0.04
malE Trx^a^(Concentration 2.5 mg/ml)	18±0.14	68.4±0.05

The protein concentration was 0.2 mg/ml, unless mentioned otherwise.

a Since thermal unfolding is irreversible for this protein, these thermodynamic parameters are apparent values.

Enthalpies are calorimetric enthalpies.

### Refolding in Buffer

**Figure 7:**
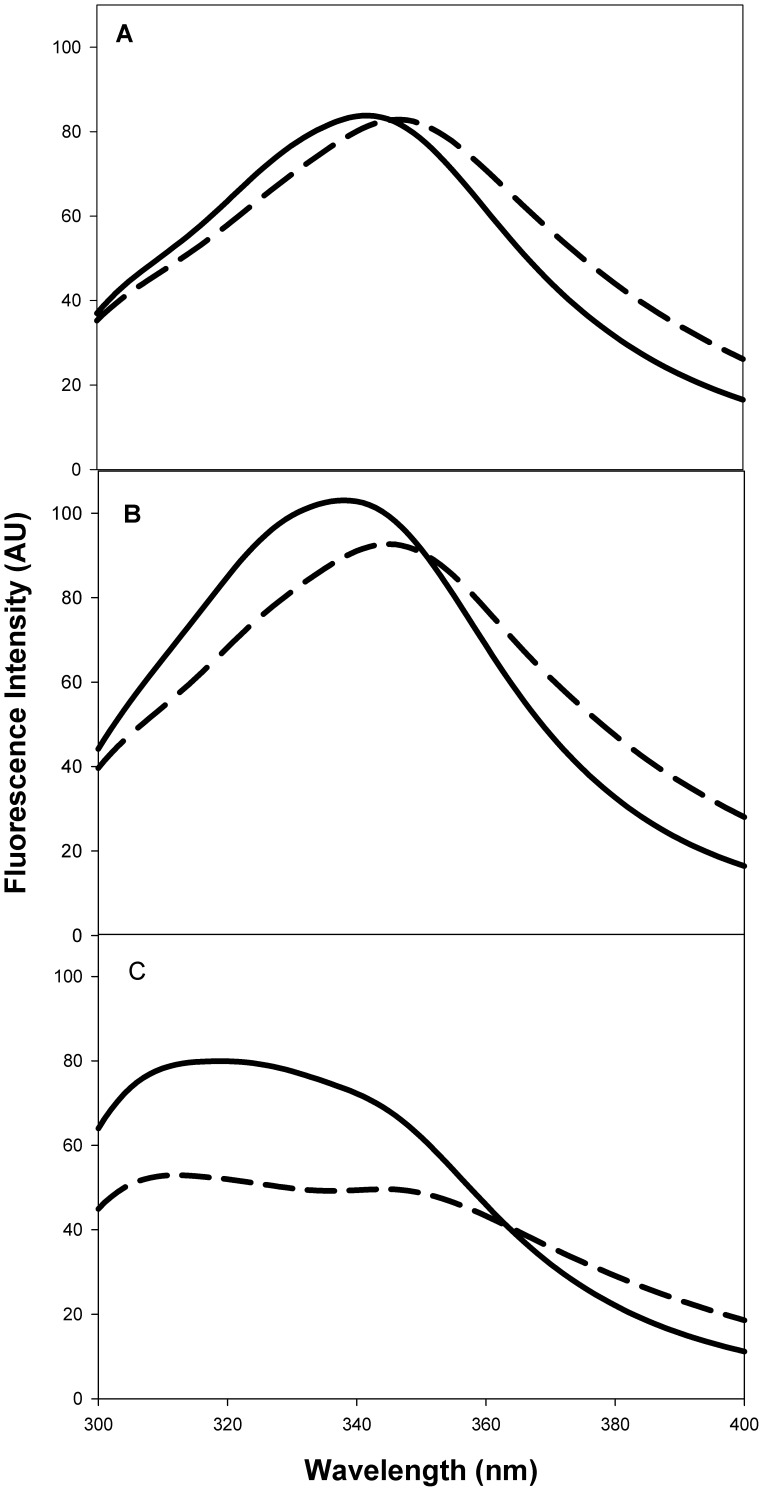
Fluorescence spectra of native (−) and refolded (–) proteins. The final protein concentrations were 5 µM at pH 7.4 in CGH-10 buffer at 25°C. (A) WT Trx, (B) pelB Trx and (C) malE Trx, respectively. Refolding of WT and pelB Trx is reversible but malE Trx refolding is poorly reversible under these conditions.

CD spectra before and after refolding were virtually identical for all three proteins (data not shown). Fluorescence spectra for WT Trx and pelB Trx, before and after refolding were similar (Figure 7). However, for malE Trx there was an appreciable decrease in the intensity of peak for the refolded protein (Figure 7). The fraction refolded was 95, 88 and 68 percent for WT Trx, pelB Trx and malE Trx respectively.

### Refolding in the Presence of Crowding Agent

**Figure 8:**
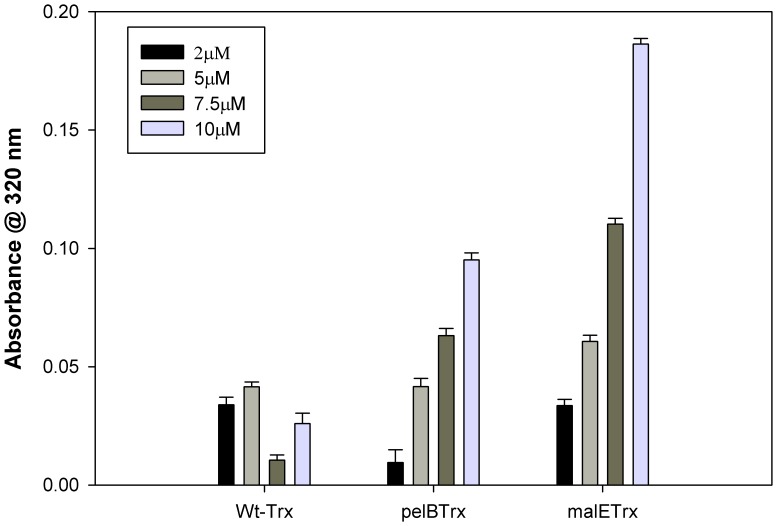
Effect of crowding agent (30% Ficoll) on Trx refolding. 100 µM protein was denatured in 4 M GdmCl (CGH-10 buffer, pH 7.4), diluted and refolded into Ficoll containing buffer. Protein aggregation was monitored using the apparent absorbance at 320 nm. Bar graphs from left to right show data for proteins refolded at final protein concentrations of 2, 5, 7.5 and 10 µM each for WT Trx, pelB Trx and malE Trx respectively.

The synthetic polysaccharide Ficoll 70 [54] is used to mimic intracellular crowded environment *in vitro*, since it is inert, highly soluble and has an average molecular mass of ∼70 kDa. The refolding efficiency of the proteins in the presence of Ficoll 70 was determined by measuring the aggregation propensities of the proteins by following absorbance at 320 nm during refolding. Aggregation was not observed when the proteins were refolded in buffer lacking Ficoll. However, in the presence of Ficoll-70 (30%), both malE Trx and to a lesser extent pelB Trx were prone to aggregation (Figure 8). This shows that signal peptide increases the propensity of thioredoxin towards aggregation while refolding.

### Insulin Reduction Assay

**Figure 9:**
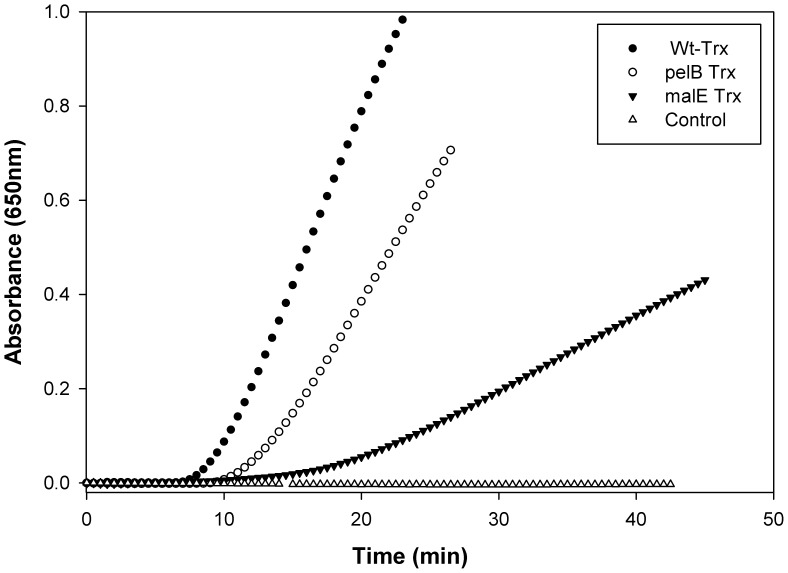
Insulin reduction assay for redox activity. Insulin aggregation following reduction was monitored by the increase in light scattering at 650 nm. Assay conditions were 0.1 M phosphate buffer, 2 mM EDTA, 0.13 mM porcine insulin, 0.33 mM DTT, and 5 µM protein. Protein identities are adjacent to each trace. Incubation mixture without protein served as negative control and WT Trx served as a positive control.

Thioredoxin A is an oxidoreductase that has previously been shown to catalyze the reduction of insulin disulfides by DTT [44]. A quantitative assay was developed which measures the rate of insulin reduction spectrophotometrically at 650 nm as turbidity formation from free insulin B chain [44]. Thioredoxin at 5 µM concentration accelerated the reaction between 0.13 mM insulin and 0.33 mM DTT. malE Trx showed a marked decrease in insulin reduction activity while pelB Trx showed intermediate activity relative to WT Trx (Figure 9). This suggest that the presence of the hydrophobic signal peptide interferes with protein activity, even though the signal peptide is accessible to proteases and does not perturb the secondary structure of the protein.

### Prediction of Aggregation Propensities of pelB and malE Leader Sequences

**Figure 10:**
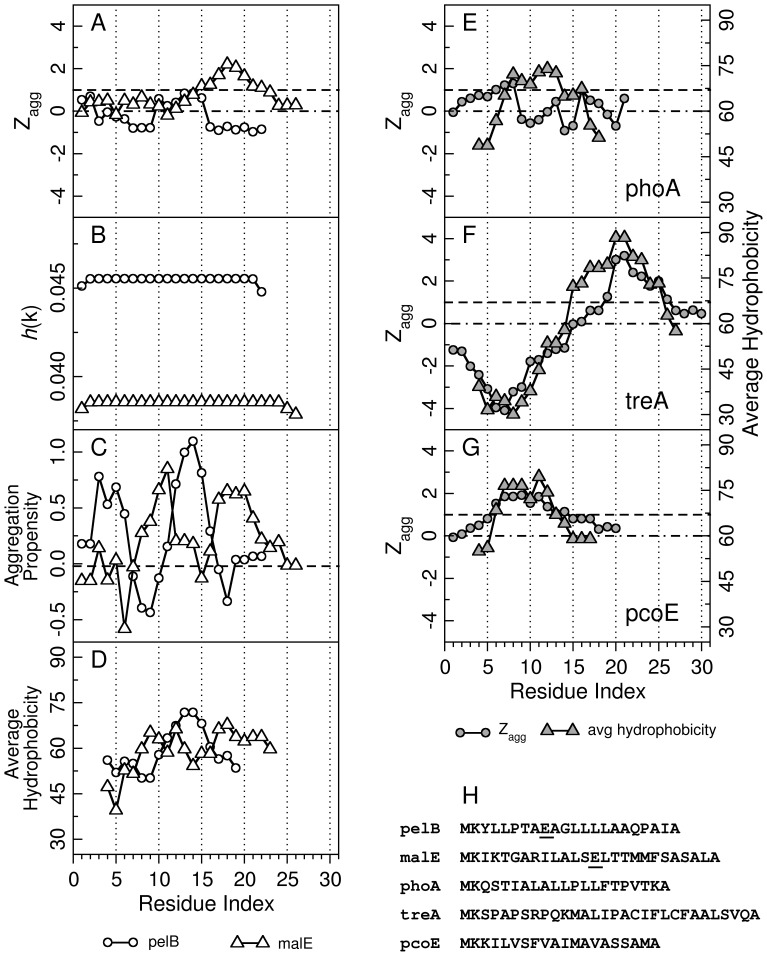
Aggregation propensity profiles of various signal peptides. Panels A–C show aggregation propensity profiles for pelB (empty circles,○) and malE (empty triangles,▵) sequences calculated using (A) Zyggregator, (B) PASTA, (C) AGGRESCAN. (D) Average hydrophobicity calculated using PREDBUR. Amino acid regions with Z_agg_>1 are considered to be aggregation prone, whereas regions with Z_agg_<0 were assumed to have low aggregation propensities. These upper and lower cut-offs are indicated by dashed line (–) and by dash-dot lines (–) respectively in panels A,E,F,G. The regions with aggregation propensity values above −0.02 are considered as hot-spots for aggregation by AGGRESCAN algorithm. The cut-off value is indicated by a dashed line (–) in C. Panel E-G show aggregation propensity profiles calculated using Zyggregator and average hydrophobicity calculated using PREDBUR for three previously studied soluble Trx fusion systems with phoA, treA, and pcoE signal sequences. Here, Z_agg_ is shown in filled circles (•), and average hydrophobicity is indicated with filled triangles (▴). The amino acid sequences for all the signal peptides are given in H. The locations of the AE mutation in pelB and malE are underlined.

The average hydrophobicites of the leader sequences were calculated with a sliding window size of 7 as described previously [48]. Plots of average hydrophobicity along the protein sequence have been used to predict the locations of buried and exposed regions. As shown in Figure 10, both malE (residues 7–14 and 16–20) and pelB (residues 9–17) show similar hydrophobicity profiles. Hence, using the hydrophobicity index it is difficult to infer that the malE leader sequence is more hydrophobic than pelB. In an alternative approach we calculated aggregation propensities using the Zyggregator [45], PASTA [46] and AGGRESCAN [47] algorithms. The Z_agg_ score for pelB and malE sequence were computed from the Zyggregator server (http://www-vendruscolo.ch.cam.ac.uk/zyggregator.php). For malE Trx the intrinsic aggregation propensity profile Z_agg_ reveals one region of high aggregation propensity (residues 15–22, Figure 10). In contrast pelB Trx, does not exhibit any region above Z_agg_ = 1. This result is consistent with the observation that the malE sequence can be more aggregation prone as compared to pelB. The PASTA and AGGRESCAN algorithms did not show any clear differences between aggregation propensities of the pelB and malE signal sequences. The AGGRESCAN calculations were also repeated for the entire protein. Here too, no substantial difference was observed between malE Trx and pelB Trx (Figure S2). As a further test we examined the predictions of the Zyggregator and average hydrophobicity algorithms for three other signal sequences, phoA, treA, and pcoE. Previous studies have shown that Trx fusions to each of these signal sequences are soluble when expressed in *E. coli*. However the fusions are not efficiently translocated to the periplasm [55], [56]. As can be seen from Figure 10, for treA and pcoE signal sequences both procedures give very similar results. However, for phoA, the Zyggregator shows a somewhat lower overall aggregation propensity than the average hydrophobicity calculations. For treA, Zyggregator predicts the C-terminal region of the sequence to have high aggregation propensity. However, this may be offset by the very low aggregation propensity of the N-terminal half of the sequence. For pcoE, the overall Z_agg_ profile is similar to that of malE, yet malE Trx is insoluble while pcoE Trx is soluble when expressed in *E. coli.* The overall conclusion from the above analysis on a limited number of sequences is that of the various approaches for predicting aggregation propensity of signal sequences, Zyggregator does better than the other three programs, though there is still only partial agreement with experimental results. One caveat to the above analysis is that it assumes the signal peptide self-aggregates. However, it is likely that aggregation during folding/unfolding is mediated by interactions between the signal sequence and other hydrophobic stretches that are exposed in the unfolded or intermediate states.

## Discussion

Signal peptide as a target recognition motif plays a key role in protein translocation and secretion [57], [58]. However, its role in directly modulating the properties of pre-proteins and hence in export is not well characterized. In the present study, we compared the effects of two different signal peptides on the folding and stability of thioredoxin relative to WT Trx lacking signal sequence. In such a case, differences in the properties of the three proteins can solely be attributed to the effect of signal peptide on the protein. The current work provides a detailed comparison among WT Trx, pelB Trx and malE Trx in terms of their thermodynamic stabilities, aggregation propensities and activities.

WT Trx and pelB Trx were isolated as soluble proteins from the periplasm while malE Trx was refolded from inclusion bodies, which is an indication of significant destabilization of malE Trx relative to pelB Trx.

ANS is a dye which binds to exposed hydrophobic patches and is used to identify partially folded protein [42]. ANS binding studies with both pelB Trx and malE Trx (Figure 3) indicate that signal peptide fusion does not result in molten globule formation.

Gel filtration studies under denaturing conditions did not show any appreciable differences in elution volume amongst the three proteins. However, given the conformational diversity and dynamic nature of the denatured state, it may be difficult to detect transient interactions between signal peptide and the rest of the protein using this technique. Under native conditions, both malE Trx and pelB Trx eluted slightly before WT Trx, consistent with the higher molecular weights of these two proteins.

The resistance of Trx to various proteases used in the current study is consistent with the earlier proteolytic digestion studies of preMBP and MBP which showed rapid removal of signal peptide to yield mature protein [49]. Proteolysis of pelB Trx and malE Trx results in rapid digestion of the signal peptide, suggesting that it is accessible to protease and has only transient interactions with the rest of the protein in the native state. Despite this, both signal peptides affect the stability and aggregation propensity with malE showing larger effects than pelB. pelB Trx shows ΔC_m_ and ΔT_m_ values of −0.1 M and −6°C relative to WT Trx. The malE Trx shows low reversibility of unfolding for both chemical and thermal denaturation, with apparent ΔC_m_ and ΔT_m_ values of −0.4 M and −15°C respectively relative to WT Trx. These data strongly suggest that malE Trx is substantially destabilized relative to pelB Trx and WT Trx. The molecule is also much more aggregation prone.

All three proteins at 10 µM final concentration can be refolded after chemical denaturation in buffer though the reversibility decreases in the order WT Trx>pelB Trx>malE Trx. In the presence of the crowding agent Ficoll-70, WT Trx still refolds without aggregation. In contrast, both malE Trx and to a lesser extent pelB Trx show a tendency to aggregate in a concentration dependent manner. This is consistent with malE Trx being targeted to inclusion bodies *in vivo.* The redox activity of three proteins also decreases in the same order.

Overall, the data suggest that the presence and identity of the signal peptide can modulate thioredoxin stability, aggregation propensity and activity. We have previously shown that the malE signal peptide results in thermodynamic destabilization of its fusion partner, MBP [49] by about 2–6 kcal/mol. Since the signal sequence did not appear to affect the structure of the native state, it was suggested that it might stabilize the unfolded state through hydrophobic interactions. In the present study, the pelB signal sequence led to a decrease in ΔG° of unfolding of Trx by ∼0.2 kcal/mol (Figure 5) and a decrease of 6°C in T_m_. For malE Trx it was not possible to get accurate thermodynamic data because of the low reversibility thermal denaturation, although there was a substantial decrease in the apparent T_m_ by 15°C and a decrease in ΔG° of unfolding by ∼1.4 kcal/mol with respect to WT Trx. It therefore appears that upon either partial or complete unfolding, the malE signal sequence causes destabilization and irreversible aggregation of the unfolded protein.

In *E. coli*, proteins are exported to the periplasm by the secretory (*sec*) pathway, which requires them to be in a translocation competent unfolded state to pass through the membrane-embedded secretion machinery [59]. However, many of these proteins are exported only after much of the polypeptide chain has been synthesized (i.e., post-translationally) [28]. The chaperone SecB in *E. coli* specifically recognizes a subset of proteins and holds them in an unfolded state until they are transferred to the rest of the secretion machinery [60].

In earlier studies, efforts were made to efficiently export *E. coli* thioredoxin to the periplasm by a post-translational pathway [29], [55] by attaching the post-translational alkaline phosphatase (PhoA) signal sequence to the thioredoxin N-terminus. However, this results in a very small amount of the protein reaching the periplasm. The poor export of thioredoxin is thought to be due to rapid folding of the protein in the cytoplasm, preventing its post-translational translocation across the cytoplasmic membrane [29], [61]. Thioredoxin is known to be efficiently exported by the co-translational pathway, when fused to appropriate signal peptides such as dsbA [29]. Thioredoxin fusions with various signal peptides have been used to identify peptides that promote co-translational translocation [55], [56]. It has been assumed when Trx fusions are not translocated, this is because of rapid folding of the thioredoxin in the cytoplasm. Consequently, the corresponding signal sequences are believed to mediate SecB dependent post-translational translocation rather than SRP dependent co-translational translocation. The present study demonstrates that the signal peptides can potentially have profound effects on the stability and aggregation propensity of the protein, especially in crowded environments. These factors may also impair translocation. Hence lack of translocation of a signal peptide-Trx fusion can either be due to rapid folding of the Trx reporter or to aggregation of the fusion during cytoplasmic folding. Thus, besides maintaining substrates in an unfolded conformation prior to export, an important function of chaperones such as SecB, may be to prevent signal peptide mediated protein aggregation. In previous studies [14], [15], we showed that the malE signal peptide significantly affected MBP folding and aggregation. MBP is a large 370 amino acid multidomain protein with complex folding kinetics. Thioredoxin is a relatively small and stable protein with well characterized folding kinetics, making it an excellent system to understand the perturbing effects of signal peptide on protein folding and stability. Despite having similar hydrophobicity, the malE and pelB signal sequences have significantly different effects on thioredoxin activity, stability and aggregation propensity. Future studies will examine the kinetics of folding, unfolding and aggregation of the malE Trx and pelB Trx fusion proteins.

## Supporting Information

Figure S1 **ESI-MS spectra of WT Trx, pelB Trx and malE Trx.** (A)WT Trx, expected and observed masses of 11673.3 and 11672.8 Da respectively. (B) pelB Trx, expected and observed masses of 14075 and 14073.5 Da respectively. (C) malE Trx, expected and observed masses of 14571 and 14573.8 Da respectively.
(TIF)

Figure S2 **The AGGRESCAN profile for the full length proteins, pelB Trx and malE Trx.**
(TIF)

## References

[1] Pugsley AP (1993) The complete general secretory pathway in gram-negative bacteria. Microbiol Rev 57: 50–108. 8096622 10.1128/mr.57.1.50-108.1993 PMC372901

[2] Stuart RA, Neupert W (2000) Making membranes in bacteria. Nature 406: 575, 577. 10.1038/35020668 10949283

[3] Cristobal S, de Gier JW, Nielsen H, von Heijne G (1999) Competition between Sec- and TAT-dependent protein translocation in Escherichia coli. EMBO J 18: 2982–2990. 10357811 10.1093/emboj/18.11.2982 PMC1171380

[4] DeLisa MP, Tullman D, Georgiou G (2003) Folding quality control in the export of proteins by the bacterial twin-arginine translocation pathway. Proc Natl Acad Sci U S A 100: 6115–6120. 12721369 10.1073/pnas.0937838100 PMC156335

[5] Izard JW, Kendall DA (1994) Signal peptides: exquisitely designed transport promoters. Mol Microbiol 13: 765–773. 7815936 10.1111/j.1365-2958.1994.tb00469.x

[6] du Plessis DJ, Nouwen N, Driessen AJ (2011) The Sec translocase. Biochim Biophys Acta 1808: 851–865. 20801097 10.1016/j.bbamem.2010.08.016

[7] Hartl FU, Lecker S, Schiebel E, Hendrick JP, Wickner W (1990) The binding cascade of SecB to SecA to SecY/E mediates preprotein targeting to the E. coli plasma membrane. Cell 63: 269–279. 2170023 10.1016/0092-8674(90)90160-g

[8] Hoffschulte HK, Drees B, Muller M (1994) Identification of a soluble SecA/SecB complex by means of a subfractionated cell-free export system. J Biol Chem 269: 12833–12839. 8175697

[9] Randall LL, Hardy SJ (1986) Correlation of competence for export with lack of tertiary structure of the mature species: a study in vivo of maltose-binding protein in E. coli. Cell 46: 921–928. 3530497 10.1016/0092-8674(86)90074-7

[10] Kusukawa N, Yura T, Ueguchi C, Akiyama Y, Ito K (1989) Effects of mutations in heat-shock genes groES and groEL on protein export in Escherichia coli. EMBO J 8: 3517–3521. 2573517 10.1002/j.1460-2075.1989.tb08517.x PMC401509

[11] Wild J, Altman E, Yura T, Gross CA (1992) DnaK and DnaJ heat shock proteins participate in protein export in Escherichia coli. Genes Dev 6: 1165–1172. 1628824 10.1101/gad.6.7.1165

[12] Randall LL, Hardy SJ (1995) High selectivity with low specificity: how SecB has solved the paradox of chaperone binding. Trends Biochem Sci 20: 65–69. 7701564 10.1016/s0968-0004(00)88959-8

[13] Francetic O, Kumamoto CA (1996) Escherichia coli SecB stimulates export without maintaining export competence of ribose-binding protein signal sequence mutants. J Bacteriol 178: 5954–5959. 8830692 10.1128/jb.178.20.5954-5959.1996 PMC178452

[14] Krishnan B, Kulothungan SR, Patra AK, Udgaonkar JB, Varadarajan R (2009) SecB-mediated protein export need not occur via kinetic partitioning. J Mol Biol 385: 1243–1256. 19028503 10.1016/j.jmb.2008.10.094

[15] Kulothungan SR, Das M, Johnson M, Ganesh C, Varadarajan R (2009) Effect of crowding agents, signal peptide, and chaperone SecB on the folding and aggregation of E. coli maltose binding protein. Langmuir 25: 6637–6648. 19358587 10.1021/la900198h

[16] Zalucki YM, Jones CE, Ng PS, Schulz BL, Jennings MP (2010) Signal sequence non-optimal codons are required for the correct folding of mature maltose binding protein. Biochim Biophys Acta 1798: 1244–1249. 20230779 10.1016/j.bbamem.2010.03.010

[17] Zalucki YM, Shafer WM, Jennings MP (2011) Directed evolution of efficient secretion in the SRP-dependent export of TolB. Biochim Biophys Acta 1808: 2544–2550. 21699884 10.1016/j.bbamem.2011.06.004 PMC3289050

[18] Robbens J, Raeymaekers A, Steidler L, Fiers W, Remaut E (1995) Production of soluble and active recombinant murine interleukin-2 in Escherichia coli: high level expression, Kil-induced release, and purification. Protein Expr Purif 6: 481–486. 8527934 10.1006/prep.1995.1064

[19] Wan EW, Baneyx F (1998) TolAIII co-overexpression facilitates the recovery of periplasmic recombinant proteins into the growth medium of Escherichia coli. Protein Expr Purif 14: 13–22. 9758746 10.1006/prep.1998.0941

[20] Schatz PJ, Beckwith J (1990) Genetic analysis of protein export in Escherichia coli. Annu Rev Genet 24: 215–248. 2088168 10.1146/annurev.ge.24.120190.001243

[21] Walter P, Johnson AE (1994) Signal sequence recognition and protein targeting to the endoplasmic reticulum membrane. Annu Rev Cell Biol 10: 87–119. 7888184 10.1146/annurev.cb.10.110194.000511

[22] von Heijne G (1985) Signal sequences. The limits of variation. J Mol Biol 184: 99–105. 4032478 10.1016/0022-2836(85)90046-4

[23] Sjostrom M, Wold S, Wieslander A, Rilfors L (1987) Signal peptide amino acid sequences in Escherichia coli contain information related to final protein localization. A multivariate data analysis. EMBO J 6: 823–831. 3556168 10.1002/j.1460-2075.1987.tb04825.x PMC553468

[24] Edman M, Jarhede T, Sjostrom M, Wieslander A (1999) Different sequence patterns in signal peptides from mycoplasmas, other gram-positive bacteria, and Escherichia coli: a multivariate data analysis. Proteins 35: 195–205. 10223292

[25] Humphreys DP, Sehdev M, Chapman AP, Ganesh R, Smith BJ, et al (2000) High-level periplasmic expression in Escherichia coli using a eukaryotic signal peptide: importance of codon usage at the 5′ end of the coding sequence. Protein Expr Purif 20: 252–264. 11049749 10.1006/prep.2000.1286

[26] Lei SP, Lin HC, Wang SS, Callaway J, Wilcox G (1987) Characterization of the Erwinia carotovora pelB gene and its product pectate lyase. J Bacteriol 169: 4379–4383. 3040692 10.1128/jb.169.9.4379-4383.1987 PMC213756

[27] Steiner D, Forrer P, Stumpp MT, Pluckthun A (2006) Signal sequences directing cotranslational translocation expand the range of proteins amenable to phage display. Nat Biotechnol 24: 823–831. 16823375 10.1038/nbt1218

[28] Josefsson LG, Randall LL (1983) Analysis of cotranslational proteolytic processing of nascent chains using two-dimensional gel electrophoresis. Methods Enzymol 97: 77–85. 6361484 10.1016/0076-6879(83)97121-5

[29] Schierle CF, Berkmen M, Huber D, Kumamoto C, Boyd D, et al (2003) The DsbA signal sequence directs efficient, cotranslational export of passenger proteins to the Escherichia coli periplasm via the signal recognition particle pathway. J Bacteriol 185: 5706–5713. 13129941 10.1128/JB.185.19.5706-5713.2003 PMC193964

[30] Holmgren A (1968) Thioredoxin. 6. The amino acid sequence of the protein from escherichia coli B. Eur J Biochem 6: 475–484. 4883076 10.1111/j.1432-1033.1968.tb00470.x

[31] Eklund H, Gleason FK, Holmgren A (1991) Structural and functional relations among thioredoxins of different species. Proteins 11: 13–28. 1961698 10.1002/prot.340110103

[32] Katti SK, LeMaster DM, Eklund H (1990) Crystal structure of thioredoxin from Escherichia coli at 1.68 A resolution. J Mol Biol 212: 167–184. 2181145 10.1016/0022-2836(90)90313-B

[33] Jeng MF, Campbell AP, Begley T, Holmgren A, Case DA, et al (1994) High-resolution solution structures of oxidized and reduced Escherichia coli thioredoxin. Structure 2: 853–868. 7812718 10.1016/s0969-2126(94)00086-7

[34] Slaby I, Cerna V, Jeng MF, Dyson HJ, Holmgren A (1996) Replacement of Trp28 in Escherichia coli thioredoxin by site-directed mutagenesis affects thermodynamic stability but not function. J Biol Chem 271: 3091–3096. 8621706 10.1074/jbc.271.6.3091

[35] Holmgren A (1972) Tryptophan fluorescence study of conformational transitions of the oxidized and reduced form of thioredoxin. J Biol Chem 247: 1992–1998. 4552684

[36] Kelley RF, Stellwagen E (1984) Conformational transitions of thioredoxin in guanidine hydrochloride. Biochemistry 23: 5095–5102. 6391536 10.1021/bi00317a003

[37] Kelley RF, Shalongo W, Jagannadham MV, Stellwagen E (1987) Equilibrium and kinetic measurements of the conformational transition of reduced thioredoxin. Biochemistry 26: 1406–1411. 3552046 10.1021/bi00379a029

[38] Pedone E, Bartolucci S, Rossi M, Pierfederici FM, Scire A, et al (2003) Structural and thermal stability analysis of Escherichia coli and Alicyclobacillus acidocaldarius thioredoxin revealed a molten globule-like state in thermal denaturation pathway of the proteins: an infrared spectroscopic study. Biochem J 373: 875–883. 12733987 10.1042/BJ20021747 PMC1223541

[39] Nielsen H, Engelbrecht J, Brunak S, von Heijne G (1997) Identification of prokaryotic and eukaryotic signal peptides and prediction of their cleavage sites. Protein Eng 10: 1–6. 10.1093/protein/10.1.1 9051728

[40] Ghoshal AK, Swaminathan CP, Thomas CJ, Surolia A, Varadarajan R (1999) Thermodynamic and kinetic analysis of the Escherichia coli thioredoxin-C′ fragment complementation system. Biochem J 339 (Pt 3): 721–727. PMC1220209 10215612

[41] Ganesh C, Shah AN, Swaminathan CP, Surolia A, Varadarajan R (1997) Thermodynamic characterization of the reversible, two-state unfolding of maltose binding protein, a large two-domain protein. Biochemistry 36: 5020–5028. 9125524 10.1021/bi961967b

[42] Sheshadri S, Lingaraju GM, Varadarajan R (1999) Denaturant mediated unfolding of both native and molten globule states of maltose binding protein are accompanied by large deltaCp’s. Protein Sci 8: 1689–1695. 10452613 10.1110/ps.8.8.1689 PMC2144416

[43] Agashe VR, Udgaonkar JB (1995) Thermodynamics of denaturation of barstar: evidence for cold denaturation and evaluation of the interaction with guanidine hydrochloride. Biochemistry 34: 3286–3299. 7880824 10.1021/bi00010a019

[44] Holmgren A (1979) Thioredoxin catalyzes the reduction of insulin disulfides by dithiothreitol and dihydrolipoamide. J Biol Chem 254: 9627–9632. 385588

[45] Tartaglia GG, Vendruscolo M (2008) The Zyggregator method for predicting protein aggregation propensities. Chem Soc Rev 37: 1395–1401. 18568165 10.1039/b706784b

[46] Trovato A, Seno F, Tosatto SC (2007) The PASTA server for protein aggregation prediction. Protein Eng Des Sel 20: 521–523. 17720750 10.1093/protein/gzm042

[47] Conchillo-Sole O, de Groot NS, Aviles FX, Vendrell J, Daura X, et al (2007) AGGRESCAN: a server for the prediction and evaluation of “hot spots” of aggregation in polypeptides. BMC Bioinformatics 8: 65. 17324296 10.1186/1471-2105-8-65 PMC1828741

[48] Varadarajan R, Nagarajaram HA, Ramakrishnan C (1996) A procedure for the prediction of temperature-sensitive mutants of a globular protein based solely on the amino acid sequence. Proc Natl Acad Sci U S A 93: 13908–13913. 8943034 10.1073/pnas.93.24.13908 PMC19465

[49] Beena K, Udgaonkar JB, Varadarajan R (2004) Effect of signal peptide on the stability and folding kinetics of maltose binding protein. Biochemistry 43: 3608–3619. 15035631 10.1021/bi0360509

[50] Chakraborty K, Thakurela S, Prajapati RS, Indu S, Ali PS, et al (2005) Protein stabilization by introduction of cross-strand disulfides. Biochemistry 44: 14638–14646. 16262263 10.1021/bi050921s

[51] Woody RW (1995) Circular dichroism. Methods Enzymol 246: 34–71. 7538625 10.1016/0076-6879(95)46006-3

[52] Chakrabarti A, Srivastava S, Swaminathan CP, Surolia A, Varadarajan R (1999) Thermodynamics of replacing an alpha-helical Pro residue in the P40S mutant of Escherichia coli thioredoxin. Protein Sci 8: 2455–2459. 10595549 10.1110/ps.8.11.2455 PMC2144191

[53] Das M, Kobayashi M, Yamada Y, Sreeramulu S, Ramakrishnan C, et al (2007) Design of disulfide-linked thioredoxin dimers and multimers through analysis of crystal contacts. J Mol Biol 372: 1278–1292. 17727880 10.1016/j.jmb.2007.07.033

[54] Zimmerman SB, Minton AP (1993) Macromolecular crowding: biochemical, biophysical, and physiological consequences. Annu Rev Biophys Biomol Struct 22: 27–65. 7688609 10.1146/annurev.bb.22.060193.000331

[55] Debarbieux L, Beckwith J (1998) The reductive enzyme thioredoxin 1 acts as an oxidant when it is exported to the Escherichia coli periplasm. Proc Natl Acad Sci U S A 95: 10751–10756. 9724776 10.1073/pnas.95.18.10751 PMC27967

[56] Huber D, Boyd D, Xia Y, Olma MH, Gerstein M, et al (2005) Use of thioredoxin as a reporter to identify a subset of Escherichia coli signal sequences that promote signal recognition particle-dependent translocation. J Bacteriol 187: 2983–2991. 15838024 10.1128/JB.187.9.2983-2991.2005 PMC1082830

[57] Milstein C, Brownlee GG, Harrison TM, Mathews MB (1972) A possible precursor of immunoglobulin light chains. Nat New Biol 239: 117–120. 4507519 10.1038/newbio239117a0

[58] Blobel G, Dobberstein B (1975) Transfer of proteins across membranes. I. Presence of proteolytically processed and unprocessed nascent immunoglobulin light chains on membrane-bound ribosomes of murine myeloma. J Cell Biol 67: 835–851. 811671 10.1083/jcb.67.3.835 PMC2111658

[59] Fekkes P, Driessen AJ (1999) Protein targeting to the bacterial cytoplasmic membrane. Microbiol Mol Biol Rev 63: 161–173. 10066835 10.1128/mmbr.63.1.161-173.1999 PMC98961

[60] Hardy SJ, Randall LL (1991) A kinetic partitioning model of selective binding of nonnative proteins by the bacterial chaperone SecB. Science 251: 439–443. 1989077 10.1126/science.1989077

[61] Huber D, Cha MI, Debarbieux L, Planson AG, Cruz N, et al (2005) A selection for mutants that interfere with folding of Escherichia coli thioredoxin-1 in vivo. Proc Natl Acad Sci U S A 102: 18872–18877. 16357193 10.1073/pnas.0509583102 PMC1323206

