# Supplementary figures and images for: Effect of Signal Peptide on Stability and Folding of Escherichia coli Thioredoxin
Source: PLoS One. 2013 May 7;8(5):e63442. doi: 10.1371/journal.pone.0063442 (PMC3646739; doi:10.1371/journal.pone.0063442)

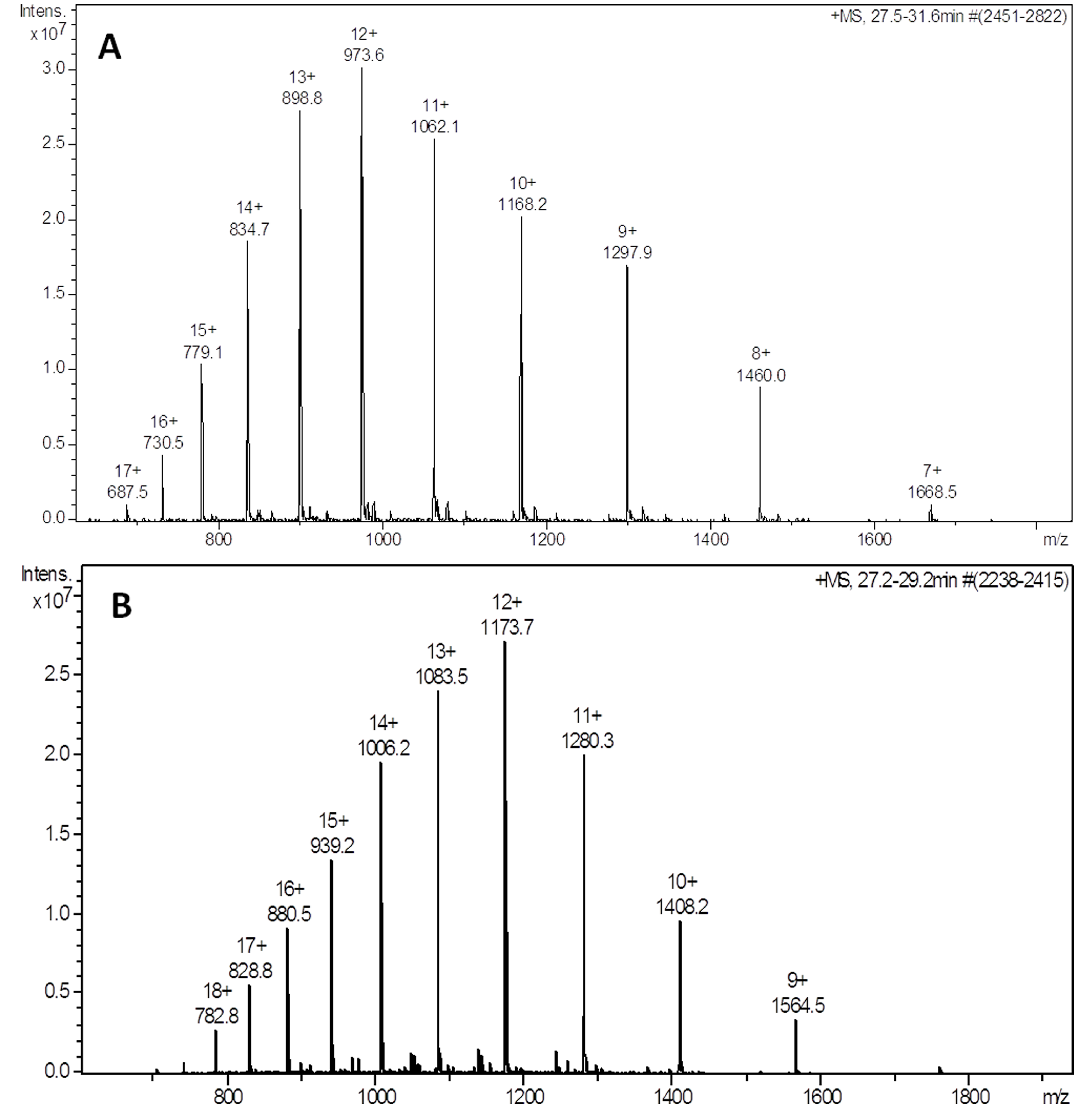

Supplement: Figure S1 — ESI-MS spectra of WT Trx, pelB Trx and malE Trx. (A)WT Trx, expected and observed masses of 11673.3 and 11672.8 Da respectively. (B) pelB Trx, expected and observed masses of 14075 and 14073.5 Da respectively. (C) malE Trx, expected and observed masses of 14571 and 14573.8 Da respectively. (TIF) [file pone.0063442.s001.tif]
